# Integrated Omics Approaches to Explore a New System of Genetic Control of Dibenzothiophene Desulfurization and Aromatic Ring Cleavage by *Gordonia alkanivorans* Strain 135

**DOI:** 10.3390/biology14020188

**Published:** 2025-02-12

**Authors:** Ekaterina Frantsuzova, Alexander Bogun, Anna Vetrova, Elizaveta Kazakova, Tomiris Kusainova, Irina Tarasova, Irina Pozdnyakova-Filatova, Yanina Delegan

**Affiliations:** 1 Institute of Biochemistry and Physiology of Microorganisms, Federal Research Center “Pushchino Scientific Center for Biological Research of Russian Academy of Sciences” (FRC PSCBR RAS), Pushchino 142290, Russia; frantsuzova.ee@gmail.com (E.F.); bogun62@mail.ru (A.B.); phdvetrova@gmail.com (A.V.); irafilatova24@gmail.com (I.P.-F.); 2 V. L. Talrose Institute for Energy Problems of Chemical Physics, N. N. Semenov Federal Research Center of Chemical Physics, Russian Academy of Sciences, Moscow 119334, Russia; kazakovaem@gmail.com (E.K.); kusainova7531@gmail.com (T.K.); iatarasova@yandex.ru (I.T.)

**Keywords:** *Gordonia*, biodegradation, aromatic compounds, transcriptomics, proteomics

## Abstract

Dibenzothiophene (DBT) is a widespread environmental pollutant. The most common metabolic pathway for DBT degradation by *Gordonia* strains is the 4S pathway, which is under the control of the *dsz* operon. The ability to utilize DBT as the sole source of sulfur in *Gordonia alkanivorans* strain 135 has been revealed. The *dsz* operon was not detected in the genome of strain 135. In this work, using genomic, transcriptomic, and proteomic data of strain 135, it was shown that an alternative pathway of DBT transformation is possible in non-*dsz Gordonia*; the *sfn*B and *tau*D genes and two acyl-dehydrogenase genes are significantly involved in the desulfurization process.

## Simple Summary

Polycyclic aromatic compounds and their substituted derivatives are hazardous environmental pollutants. This paper discusses a new pathway for the bacterial degradation of a sulfur-containing aromatic compound. The possibility of an alternative genetic control method for this process is demonstrated using *Gordonia alkanivorans* strain 135. Experimental studies based on physiological, transcriptomic, and proteomic analyses have enabled the authors to identify a set of genes involved in the degradation of dibenzothiophene.

## 1. Introduction

**Figure 1:**
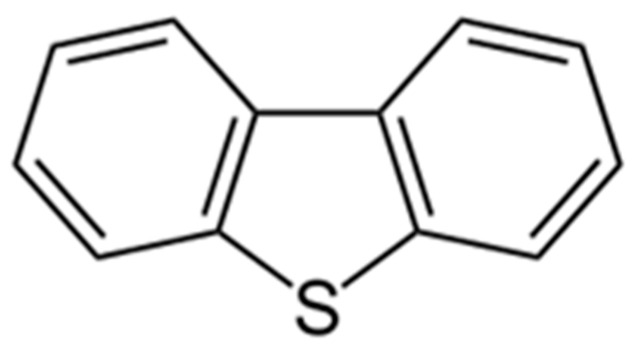
Structure of DBT.

Organic pollutants with an aromatic structure can be released into the environment either as hydrocarbons or as their derivatives, which contain amino and nitro groups, halogens, nitrogen, or sulfur as heteroatoms. Substituents often increase the toxicity of aromatic molecules [1]. The risks associated with substituted aromatic compounds were underestimated until recently [2]. Substituted aromatic compounds accumulate readily in environmental matrices such as soils, sediments, automobile exhaust, and atmospheric particles. These compounds typically exhibit low water solubility and limited biodegradability [3]. In upper soil layers, their concentrations can increase due to precipitation and/or direct inputs of crude oil [4]. Common pollutants with heteroaromatic structures include sulfur-containing compounds such as benzothiophene (BT), dibenzothiophene (DBT), and their derivatives. Among these, DBT is the most prevalent. Its chemical structure consists of two benzene rings and a central five-membered ring containing a sulfur heteroatom (Figure 1).

The ability to degrade DBT is widespread among bacteria from various taxonomic groups. Bacterial pathways for DBT catabolism can be classified into two main categories based on the role these compounds play in bacterial metabolism. The first category comprises non-desulfurizing pathways, in which DBT serves as a carbon source. In these pathways, degradation involves the destruction of the edge rings of the DBT molecule. An example of a non-desulfurizing pathway is the Kodama pathway, which is common in Gram-negative bacteria [5]. The Kodama pathway involves the oxidative cleavage of DBT through carbon–carbon bond breaking to produce hydroxyformyl-benzothiophene as the end product [6].

The second category of DBT transformation pathways includes selective desulfurization processes, where DBT serves as a sulfur source for bacterial growth. In these pathways, enzymes target the central ring of DBT. Bacterial desulfurization of DBT offers a promising and environmentally friendly approach to reducing the sulfur content in oil. This process preserves the aromatic rings of DBT, thereby preserving the calorific value of the fuel. Among bacterial desulfurization processes, the most prevalent is the 4S pathway (including its extended variants), which involves aerobic cleavage of the C-S bond, aerobic cleavage via a destructive pathway, and anaerobic C-S bond cleavage. Genetic control of the 4S pathway has been extensively studied in numerous papers. The enzyme systems responsible for biodesulfurization via the 4S pathway vary depending on the temperature adaptation ability of the bacteria. Specifically, mesophilic bacteria employ Sox, Dsz, and Mds enzymes, while thermophilic bacteria utilize Tds and Bds enzymes [7].

The 4S pathway was first described in *Rhodococcus erythropolis* strain IGTS8 (ATCC 53968) [8] and has since been identified in other members of the phylum *Actinomycetota*. The genes associated with the 4S pathway are organized in the *dsz* operon, comprising *dsz*A (encoding DBT-sulfone monooxygenase), *dsz*B (2-hydroxybiphenyl-2-sulfinate desulfinase), and *dsz*C (DBT monooxygenase), along with the freestanding *dsz*D gene, which encodes NADH-FMN oxidoreductase. The final product of the 4S pathway is 2-hydroxybiphenyl (2-HBP), which is typically accumulated at the end of the bacterial cultivation process on DBT.

DBT can be utilized as the sole sulfur source, as commonly observed in *Gordonia*. DBT-utilizing strains have been reported in several species, including *G. desulfuricans* [9], *G. amicalis* [10], *G. aichiensis* [11], *G. alkanivorans*, and *G. terrae* [12]. Numerous studies have confirmed that DBT transformation in *Gordonia* occurs via the 4S pathway, with the associated *dsz* genes located on both chromosomes and plasmids.

This paper is focused on *G. alkanivorans* strain 135, which has demonstrated the ability to utilize DBT as a sulfur source. This strain degraded 45.26% of DBT (initial concentration of 0.2 mM) [13]. Surprisingly, no *dsz* genes were identified in its genome, which is unusual for a *Gordonia* strain capable of DBT desulfurization. To date, there are no reports on *Gordonia* strains capable of utilizing DBT without *dsz* genes. The combination of two factors (the experimentally confirmed ability to utilize DBT and the absence of *dsz* genes in the genome of the strain) has led to the hypothesis that the genetic control of DBT catabolism may be organized differently in *G. alkanivorans* strain 135. Using strain 135 as an example, we hypothesized that representatives of the *Actinomycetota* phylum may have a unique mechanism of genetic control of DBT catabolism that has not been studied so far.

The aim of this study was to investigate the genetic control of the desulfurization process and the subsequent cleavage of the aromatic rings of DBT in *G. alkanivorans* strain 135. The genome analysis performed for this strain earlier [13] did not allow us to unambiguously determine the spectrum of genes involved in DBT catabolism. Thus, we did not limit ourselves to experiments on a limited spectrum of genes and used total RNA sequencing and proteomic analysis.

## 2. Materials and Methods

### 2.1. Study Object

*G. alkanivorans* strain (formerly *Gordonia* sp.) 135 was isolated in our laboratory from oil-contaminated soil (Moscow, Russia). It was deposited in the All-Russian Collection of Microorganisms under number VKM Ac-2849D.

### 2.2. Media and Cultivation Conditions

The following media were used in experiments with strain 135:

1. Evans medium [14] of the following composition (per liter): K_2_HPO_4_—8.71 g, 5 M NH_4_Cl—1 mL, 0.1 M Na_2_SO_4_—1 mL, 62 mM MgCl_2_—1 mL, 1 mM CaCl_2_—1 mL, 0.005 mM (NH_4_)_6_Mo_7_O_24_ × 4H_2_O—1 mL, trace elements—1 mL, pH 7.0. Composition of trace elements solution in 1% HCl, (g/L): ZnO—0.41, FeCl_2_ × 6H_2_O—5.4, MnCl_2_ × 4H_2_O—2.00, CuCl_2_ × 2H_2_O—0.17, CoCl_2_ × 6H_2_O—0.48, and H_3_BO_3_—0.06.

2. Sulfur-free SFM medium [15] of the following composition (per liter): NH_4_Cl—1.22 g, K_2_HPO_4_—2.5 g, Na_2_HPO_4_ × 2H_2_O—2.5 g, MgCl_2_ × 6H_2_O—0.17 g, trace elements—0.5 mL, and pH 7.5. Trace elements solution composition (per liter): EDTA—25 g, ZnCl_2_—2.136 g, MnCl_2_ × 4H_2_O—2.5 g, CoCl_2_ × 6H_2_O—0.3 g, CuCl_2_ × 2H_2_O—0.2 g, NaMoO_4_ × 2H_2_O—0.4 g, CaCl_2_ × 2H_2_O—4.5 g, FeCl_2_ × 6H_2_O—2.9 g, H_3_BO_3_—1.0 g, and KI—0.1 g.

3. Agarized medium Luria Broth (LB) [16], with the following composition (per liter): agar—20 g, bacto-tryptone—10 g, yeast extract—5 g (all from “Difco”, Sparks, MD, USA), and NaCl—10 g.

Agarized LB medium was used to check the purity of the culture.

Samples for transcriptomic and proteomic analysis were prepared in three biological replicates in 750 mL Erlenmeyer flasks containing 200 mL of medium and were incubated at 37 °C for 5 days in an orbital shaker at 180 rpm [13]. For the control condition, cultures were grown in liquid Evans medium, where sulfate served as the sole sulfur source and glucose as the carbon source. For the experimental condition, cultures were grown in liquid sulfur-free SFM medium, supplemented with a 0.1M solution of DBT in DMFA as the sulfur source and glucose as the carbon source. The final concentration of DBT was 37 mg/L. After incubation, cells were pelleted via centrifugation at 10,000 rpm for 5 min.

### 2.3. Sample Preparation, cDNA Library Construction, and Transcriptome Sequencing

Total RNA was extracted using the Aurum Total RNA Mini Kit (Bio-Rad, Hercules, CA, USA), and the quality of the RNA preparations was assessed using agarose gel electrophoresis. Ribosomal RNA was removed with the Ribo-Zero rRNA Removal Kit (Epicentre Biotechnologies, Madison, WI, USA). RNA concentrations were measured spectrophotometrically using a NanoDrop ND-2000 spectrophotometer (NanoDrop Technologies, Wilmington, DE, USA). Paired-end sequencing of the cDNA library was performed on an Illumina HiSeq 2000 system at Biospark (Moscow, Russia).

### 2.4. Transcriptome Assembly

The transcriptome was assembled using the previously assembled genome of *G. alkanivorans* strain 135 as the reference [17]. Read quality was evaluated with FastQC software v0.12.0 (https://www.bioinformatics.babraham.ac.uk/projects/fastqc/, accessed on 10 November 2024), and raw reads were filtered to remove low-quality reads (Q < 10), short reads, and adapter sequences using Trimmomatic v0.38 [18]. Reads were mapped to the genome with Bowtie2 v2.3.5.1 [19] and processed with Cufflinks v2.2.1 [20].

### 2.5. Differential Expression Analysis of Genes Involved in DBT Degradation

Differential expression analysis was performed using Cuffdiff [21] to identify loci with statistically significant differences in expression levels. The loci were sorted, filtered by *p*-value, and annotated by matching each locus to the corresponding ORF in the genomic DNA. Genes with a *p*-value ≤ 0.05 and a log2(fold change) ≥ 1 were considered differentially expressed genes (DEGs). FPKM values (fragments per kilobase of transcript per million mapped reads) were used for normalization and comparison between experiments. Functional annotation of transcripts was performed using the eggNOG-mapper service [22] and the Kyoto Encyclopedia of Genes and Genomes (KEGG) database [23]. The results were visualized using CummeRbund software v2.7.2 [24].

### 2.6. Validation of the Data Obtained Using RT-qPCR

Specific primers for RT-qPCR were designed using the Primer-BLAST tool (Table S1). The amplification efficiency was determined through a series of tenfold dilutions of the DNA template, and reaction specificity was confirmed via agarose gel electrophoresis. The first strand of cDNA was synthesized using a RevertAid RT Reverse Transcription Kit (Thermo Fisher Scientific, San Jose, CA, USA). Amplification reactions were performed using a PCR reagent kit with SYBR Green I dye (Syntol, Moscow, Russia) under the following program: (1) 3 min at 95 °C; (2) 20 s at 95 °C; (3) 20 s at 60 °C; (4) 5 s at 72 °C. Steps 2–4 were repeated for 40 cycles. Genomic DNA contamination in all analyzed samples did not exceed 3%. All experiments were conducted in six biological replicates. The relative quantity of mRNA was calculated using the Pfaffl method [25], and statistical analysis was performed in RStudio (version 4.0.0). Differences between the means of two independent groups were evaluated using Student’s t-test for independent samples.

### 2.7. Sample Preparation for MS1-Based Proteomics

For protein extraction, cells were lysed in 150 μL of buffer containing 2% SDS and 100 mM DTT in 20 mM Tris-HCl pH 8 and incubated at 95 °C (500 rpm) for 20 min [26]. After incubation, two steel beads (5 mm diameter; Qiagen, New York, NY, USA) were added to each sample. Lysis was performed in two iterations of the following procedures: incubation at −80 °C for 10 min, incubation at 95 °C (500 rpm) for 10 min, and mechanical disruption using a CryoMill (Retsch GmbH, Berlin, Germany) for 10 min at 30 Hz. After lysis, the samples were centrifuged at 20,000 rcf for 10 min, and the resulting supernatant was collected. The proteins were then precipitated using chloroform–methanol extraction, and the resulting pellet was dissolved in 200 μL of 1 M urea. Concentrations of proteins were measured using a Pierce BCA Protein Assay Kit (Thermo Fisher Scientific GmbH, Dreieich, Germany). Protein disulfide bonds were reduced with 10 mM dithiothreitol (neoFroxx GmbH, Einhausen, Germany) and alkylated with 15 mM iodoacetamide (Sigma-Aldrich, Saint Louis, MO, USA). The resulting protein preparations were incubated for 18 h with trypsin (Promega, Madison, WI, USA), which was added at a 1:50 (*w*/*w*) ratio. Enzymatic digestion was terminated by the addition of TFA, 1% *v*/*v* (Pallav Chemicals, Mumbai, India), to the samples. The samples were then desalted using Copure® C18 SPE cartridges (Biocomma Limited, Shenzhen, China), dried using a vacuum concentrator (Eppendorf Concentrator Plus) (Eppendorf, Hamburg, Germany), and stored at − 80 °C until LC-MS analysis.

### 2.8. LC-MS1 Data Acquisition

LC-MS experiments were performed using an Orbitrap Q Exactive HF-X mass spectrometer (Thermo Fisher Scientific, San Jose, CA, USA) coupled to an Dionex UltiMate 3000 RSLCnano system (Thermo Fisher Scientific, Germering, Germany). The short LC gradient method was implemented as described in [27]. A μ-Precolumn C18 PepMap100 trap column (5 μm, 100 Å, 300 μm i.d., 5 mm length) (Thermo Fisher Scientific, San Jose, CA, USA) and a Peaky capillary reversed-phase column (Reprosil-Pur 1.9 μm C18 AQ, 75 μm i.d., 5 cm length) (Molecta, Moscow, Russia) were employed for separation. The mobile phases were as follows: (A) 0.1% formic acid (FA) in water; (B) 80% ACN and 0.1% FA in water. The gradient was from 5% to 20% phase B in 0.5 min and from 20% to 40% 5 min at 1.5 μL/min. The total duration of the method, including column washing and equilibration, was 7.8 min. Data acquisition was performed in MS1-only mode. Samples were resuspended in water, and quantities of 1 ug were loaded for each injection.

### 2.9. Proteomic Data Processing

Peptide features were detected using Biosaur2 v0.2.16 software [28], and the proteomic search engine ms1searchpy v2.7.3 [29] was used for protein identification. Searches were performed against the GenBank annotated database (GCA_009720185.1). The mass tolerance for precursors in all data was ±8 ppm, and the minimum number of scans was set to 3. Carbamidomethylation of cysteine residues was the fixed modification, and no variable modifications or missed cleavages were allowed in the search. The search identified 1631 proteins for samples that were cultivated with sodium sulfate (36.7% of the proteome) and 1238 proteins for samples that were cultivated with DBT (27.9% of the proteome). Differential expression analysis was performed as described earlier [30]. In brief, quantitation was performed using DirectMS1Quant [31] with the following parameters: differentially regulated proteins must satisfy Benjamini–Hochberg FDR of <0.05; the fold change (FC) threshold was two standard deviations of the log2FC distribution; and intensity normalization by 1000 quantified peptides with maximal intensities was applied. The differential expression analysis yielded 50 up-regulated proteins (expressed in the presence of DBT) and 51 down-regulated proteins (expressed in the presence of sodium sulfate). The functional activity analysis was performed using QRePS v1.2.1 [32] coupled with STRING db v12.0 [33] for functional annotation of proteins. The set of enriched GO terms with corresponding GO Score is presented in Figure S1. The annotation is publicly available at https://version-12-0.string-db.org/organism/STRG0A54UKO (accessed on 6 May 2024).

## 3. Results and Discussion

### 3.1. Growth Physiology of G. alkanivorans Strain 135 on DBT and Premises for the Hypothesis of Alternative (Non-Dsz) Genetic Control of This Process

Sulfur sources for bacteria can be broadly categorized as “preferred” or “non-preferred” [34,35]. Preferred sources include sulfates, cysteine, and thiocyanate, and their presence in the cultivation medium inhibits the degradation of “non-preferred” compounds such as thiophenes. This necessitates performing DBT degradation experiments in sulfate-free media to avoid process redirection towards preferred sulfur sources. Many *Gordonia* strains utilized thiophene sulfur completely or almost completely (95–99%) within a maximum of 6–7 days, bringing the culture to a sulfur starvation state. For example, *G. rubripertincta* W3S5 degraded 99% of 0.2 mM DBT within 2 days [36], while *G. alkanivorans* 1B utilized 99% of 0.5 mM DBT in 7 days [37]. In contrast, *G. alkanivorans* strain 135, studied in this work, utilized only 45% of 0.2 mM DBT within 6 days [13], significantly less compared to the examples mentioned over a longer period.

The utilization of DBT by *Gordonia* strains via the 4S-pathway is characterized by the accumulation of 2-hydroxybiphenyl (2-HBP) [37,38]. Moreover, in the selective desulfurization process, where sulfur is removed from the central ring without affecting the edge rings, hydroxylated biphenyls (specifically 2-HBP) are the only possible end products. Maximum accumulation of 2-HBP typically occurs when DBT is completely depleted from the system and the culture enters the stationary growth phase. 2-HBP is toxic to cells, and its increased concentration in the culture medium leads to rapid cell death during the stationary phase [39]. However, it has been previously shown [13] that no accumulation of 2-HBP was observed in the culture medium when *G. alkanivorans* strain 135 was grown on DBT, with the culture remaining in the stationary phase for a prolonged period. The absence of hydroxylated biphenyl accumulation in the culture medium of *G. alkanivorans* 135 may be related to its ability to utilize hydroxylated aromatic compounds (such as phenol and catechol).

**Figure 2:**
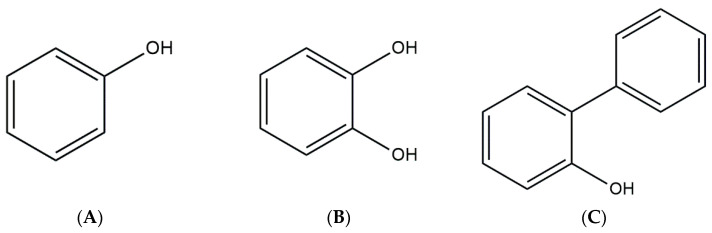
Structure of: (**A**) phenol; (**B**) catechol; (**C**) 2-HBP.

The ability of *G. alkanivorans* strain 135 to degrade aromatic compounds as carbon sources is quite unique. It has been established that mono- and polyaromatic hydrocarbons are not available for utilization by this strain, nor by any other known strain of *G. alkanivorans* species. However, it would be inaccurate to claim that the strain is completely incapable of utilizing aromatic structures. We observed growth of the strain on phenol and catechol (Figure 2), indicating its ability to cleave aromatic rings.

Based on the above observations regarding the development of the periodic culture of strain 135 when grown on DBT (absence of 2-HBP accumulation, prolonged stationary phase, and lower DBT degradation efficiency compared to 4S strains), a hypothesis was formulated that the DBT degradation process in strain 135 cells occurs differently and may be genetically organized in a manner distinct from desulfurization and subsequent breakdown of DBT aromatic rings in previously known strains. To confirm this hypothesis, transcriptomic and proteomic analysis data were utilized.

### 3.2. Whole-Transcriptome Sequencing of G. alkanivorans Strain 135

It is important to note that the main approach to studying the DBT desulfurization pathway and the function of the *dsz* operon is heterologous expression in *E. coli* [40,41]. The transcriptomic approach for studying the mechanism of DBT degradation by representatives of the genus *Gordonia* has not been used so far. Wang et al. [42] used a transcriptomic approach to study the processes occurring in cells of *G. terrae* strain C-6 during the degradation of benzothiophene (BT). The authors [42] noted that the BT desulfurization genes, designated as *bds* in the paper, had a low percentage of sequence similarity to the *dsz* operon; however, functionally, the *bds*A desulfinase, *bds*B FMNH2-dependent monooxygenase, and *bds*C alkansulfonate monooxygenase genes were similar to the *dsz*B, *dsz*C, and *dsz*A genes, respectively. Increased expression of *bds* genes when cultured on BT in [42] was confirmed using both total RNA sequencing and RT-qPCR.

We used the genome of *G. alkanivorans* strain 135 (NCBI RefSeq assembly number GCF_009720185.1) as a reference for transcriptome analysis. The genome is completely assembled and consists of a circular chromosome (size 5,039,827 bp) and a circular plasmid pG135 (size 164,963 bp) [17]. The genome assembly is a hybrid of ONT and Illumina data, with Illumina data coverage of 410x, which allows for obtaining a high-quality, reliable assembly. In the assembly, N50 corresponds to the chromosome size (5,039,827 bp); the checkM quality parameters were as follows: completeness 100% and contamination 0%.

The purpose of the transcriptome sequencing experiment for strain 135 was to investigate the effect of the sulfur source on the physiological state of the strain. To achieve this, the sulfur source (DBT or sulfates) was the only variable in this study, while the carbon source, temperature, and other cultivation conditions were kept constant.

RNA sequencing data assembly of *G. alkanivorans* strain 135 yielded 9408 and 8801 transcripts when cultured on DBT and sulfate, respectively. The mapping percentage of raw filtered reads to transcripts was 83% and 78% for the samples cultured on DBT and sulfate, respectively. Reads with Q > 10 were used for mapping. An attempt to map unfiltered reads per genome resulted in a lower mapping efficiency. We also tried to increase the quality threshold and use only reads with Q > 20 in the analysis. This slightly increased the mapping percentage but resulted in a lower coverage. Therefore, the quality threshold (Q > 10) was selected to balance the two factors mentioned above and thus to obtain efficient mapping while avoiding significant data loss.

**Figure 3:**
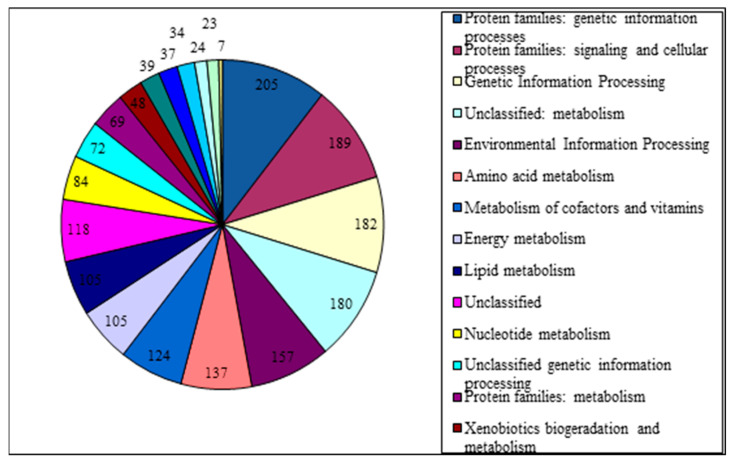
Results of functional annotation of transcripts of *G. alkanivorans* strain 135.

The reads were used to calculate log2 fold change and the differential expression of genes under different conditions. A total of 4428 transcripts were predicted, of which 2170 (49%) were annotated (Figure 3).

**Figure 4:**
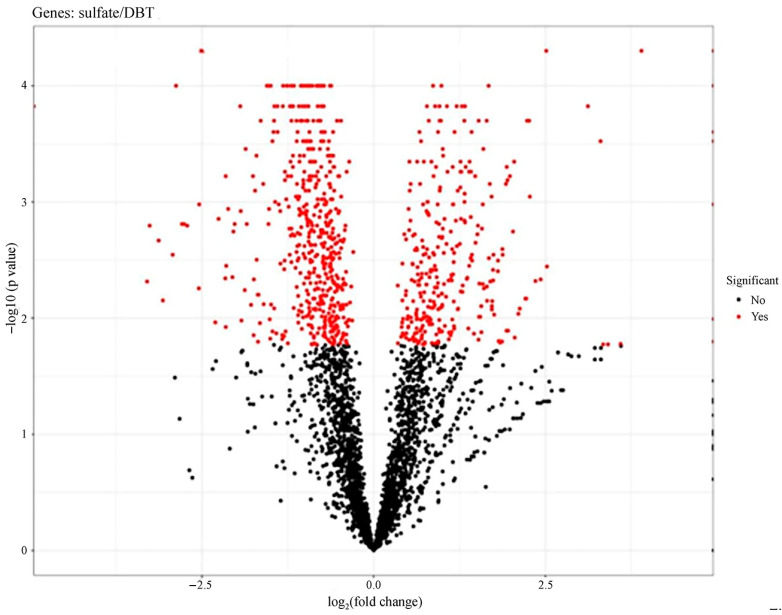
Expression profile of *G. alkanivorans* strain 135 grown with DBT or sodium sulfate (“sulfate”) as the sole sulfur source. The graph shows statistically significant genes (*p*-value < 0.05) in the upper part of the graph (low *p*-values on the *Y*-axis), with the left and right sections of the graph (*X*-axis) representing differentially expressed genes during cultivation on DBT and sodium sulfate, respectively.

Among all CDSs in the genome of strain 135, 96.4% showed no expression changes when cultured with different sulfur sources. However, 145 genes were significantly differentially expressed (log2 fold change modulus > 2, *p*-value < 0.05) (Figure 4, Tables S2 and S3).

Out of the 115 down-regulated genes, 82 genes (71%) were annotated and assigned to various functional categories. A significant portion of these 82 genes belonged to the category “Genetic Information Processes”. The subcategory “sulfur metabolism” within the “Energy metabolism” category included six genes (whose expression in the DBT sample was higher than in the sodium sulfate sample), as presented in Table 1.

The enzyme encoded by the *met*X gene catalyzes the conversion reaction of L-homoserine to O-succinyl-L-homoserine, which is one of the steps in cysteine and methionine metabolism [43]. In turn, the enzyme encoded by the *nrn*A gene is responsible for the conversion of phosphoadenylyl sulfate to adenylyl sulfate (R00508, https://www.kegg.jp/entry/R00508, accessed on 20 January 2025). We assumed that they do not play a key role in DBT catabolism and, therefore, did not include them in the spectrum of target genes.

In addition to the genes listed in Table 1, the change in expression in response to DBT was also observed in two acyl-CoA dehydrogenase genes and the *sfn*B gene, which, due to some sequence homology with *dsz*C of *G. alkanivorans* RIPI90A, were initially proposed as candidate genes involved in this process [13].

Thus, to further confirm involvement in the DBT catabolism process (based on RNA sequencing data analysis results (log2 fold change values) and KO annotation), seven genes were selected (Table 2).

The involvement of the genes selected through transcriptomic analysis in the DBT desulfurization process was confirmed by qRT-PCR.

### 3.3. Validation of Transcriptomic Data by qRT-PCR

**Figure 5:**
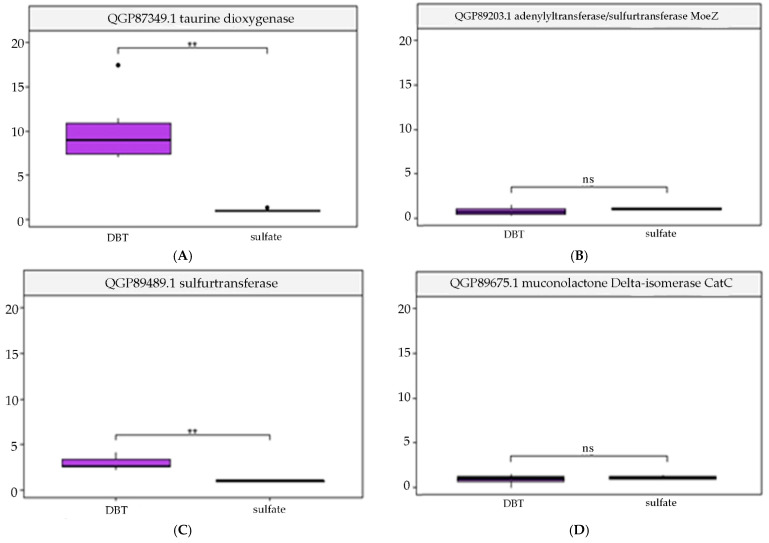

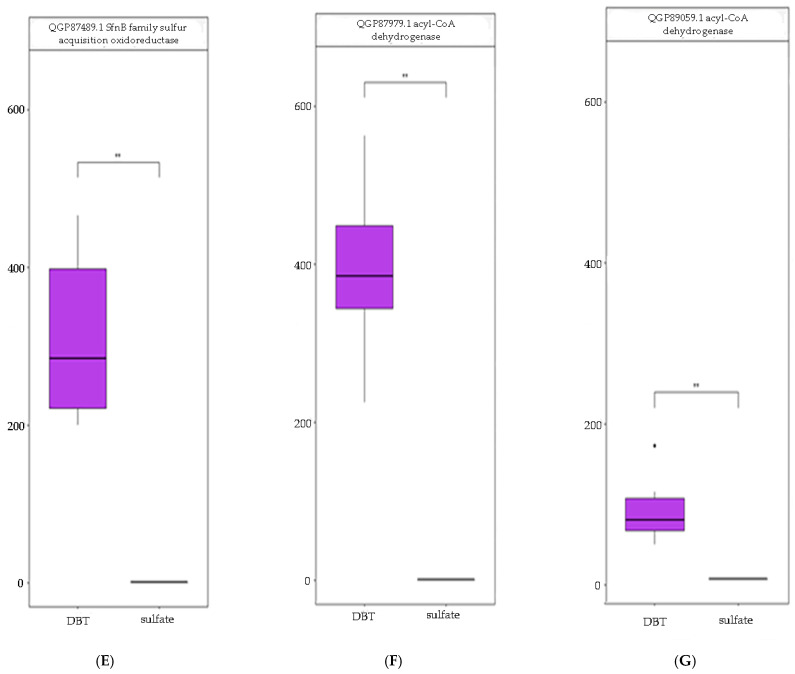
Relative mRNA abundance of genes: (**A**) taurine dioxygenase (QGP87349.1 (*tau*D)); (**B**) adenylyltransferase/sulfurtransferase (QGP89203.1 (*moe*Z)); (**C**) thiosulfate/3-mercaptopyruvate sulfurtransferase (QGP89489.1 (*sse*A)); (**D**) muconolactone delta isomerase (QGP89675.1 (*cat*C)); (**E**) sulfur acquisition oxidoreductase (QGP87489.1 (*sfn*B)); (**F**) acyl-CoA dehydrogenase (QGP87979.1 (GKZ92_RS10190)); (**G**) acyl-CoA dehydrogenase (QGP89059.1 (GKZ92_RS16320)). The Y-axis reflects the relative amount of mRNA (expressed as the fold change). “ns” means “*p* > 0.05”, “**” means “*p* ≤ 0.01”.

After cultivating cells with DBT as the sulfur source, the mRNA levels of the following genes were statistically significantly increased: QGP87349.1 taurine dioxygenase *tau*D (10.1-fold increase (*p*-value = 0.002373) (Figure 5A)), QGP89489.1 thiosulfate/3-mercaptopyruvate sulfurtransferase *sse*A (2.9-fold increase (*p*-value = 0.001287) (Figure 5C)), QGP87489.1 *sfn*B family sulfur acquisition oxidoreductase (312-fold increase (*p*-value = 0.001166) (Figure 5E)), QGP87979.1 acyl-CoA dehydrogenase (393-fold increase (*p*-value = 0.0004172) (Figure 5F)), and QGP89059.1 acyl-CoA dehydrogenase (89-fold increase (*p*-value = 0.004969) (Figure 5G)).

Thus, it was established that the presence of DBT in the cultivation medium as a sulfur source significantly alters the physiological state of the following genes: *tau*D taurine dioxygenase, *sfn*B sulfur acquisition oxidoreductase, and two acyl-CoA dehydrogenases.

### 3.4. Hypotheses About the Role of tauD, sfnB, and Two Acyl-CoA Dehydrogenase Genes in the DBT Degradation Process in Gordonia alkanivorans 135

As mentioned earlier, sulfur sources for bacteria can be conventionally divided into “preferred” or “non-preferred” categories. Preferred sources include sulfates, while non-preferred sources include DBT. According to [44], in the absence of sufficient inorganic sulfur, *Rhodococcus* strains switch to non-preferred sources to meet their sulfur requirements. The switch between preferred and non-preferred sulfur sources suggests that assimilation processes proceed via sulfur-containing organic compounds that contain C-S bonds [45].

The operons *tau*ABCD and *ssu*EADCB code for the taurine and alkanesulfonate utilization systems, respectively [46].

Taurine and alkanesulfonate are transformed into sulfite by taurine dioxygenase TauD and monooxygenase SsuD, respectively [47]. The gene *ssu*D belongs to the two-component FMNH2-dependent family of monooxygenases and participates in the cleavage of the C-S bond. SsuD is involved in bacterial desulfurization of sulfonates in both Gram-negative and -positive bacteria [48,49,50]. Ahmad et al. [51] suggested that DBT is first converted into a sulfonate through the action of DszC- and DszA-like enzymes, and then, via Ssu group clusters, the organic sulfur is converted into inorganic sulfur. As observed in our study, no analogs of DszB desulfinase were found in the genome of the strain, while *dsz*C- and *dsz*A-like genes were present [52].

There are no reports in the literature regarding the response of taurine dioxygenase (indicated by the increased expression of the *tau*D gene) to the presence of DBT in cultivation media. In [42], an increase in the expression of taurine and alkanesulfonate binding and transport systems was observed in the *G. terrae* strain when grown on BT. Given that both DBT and BT are difficult-to-utilize, non-preferred sulfur sources, it is possible that the increased expression of these systems represents an attempt by these strains to overcome sulfur limitations (caused by the limited availability of sulfur in DBT and BT) by activating genetic sulfur utilization systems from preferred sources, should they be present in the cultivation medium.

The most pronounced difference in expression in response to different sulfur sources (sulfates/DBT) was observed for the *sfn*B gene and two acyl-CoA dehydrogenase genes, which confirmed the previously formulated assumptions about their involvement in the DBT degradation process. *sfn* genes are mainly known for their roles in the context of dimethyl sulfoxide and dimethyl sulfone utilization by *Pseudomonas* strains [53], where *sfn*B is an acyl-CoA dehydrogenase and *sfn*G is an FMNH2-dependent monooxygenase, similar to the *dsz*C and *dsz*A genes. *sfn* genes are widespread in *Gordonia*, as well as in *Nocardia* and *Tsukamurella*, but not in *Rhodococcus*. Analysis of the occurrence of these genes in the genomes of *G. alkanivorans* strains showed that the *dsz* and *sfn* genes are not mutually exclusive (Table S4). Some strains contain both *sfn* and *dsz*, while others contain *sfn* but lack *dsz*. However, combinations of “*dsz* present, *sfn* absent” were not found in *G. alkanivorans*; *sfn* genes are quite common in this genus. Moreover, *sfn* genes can be considered as characteristic examples of genes predominantly found in the genus *Gordonia*. Thus, globally, *sfn* genes are primarily present in *Gordonia*, in contrast to *dsz* genes.

In summary, it can be noted that the fundamental scheme of DBT degradation (sequential oxidation of the central ring and sulfur extraction) in the *G. alkanivorans* strain is similar to the processes occurring in *dsz* strains of *Gordonia*. Moreover, in terms of the chemistry of the reactions, this is the only possible pathway for sulfur extraction from DBT. Transcriptomic analysis and subsequent validation using RT-qPCR revealed how the genetic control of this process is regulated. The FMNH2-dependent monooxygenase gene *sfn*B is likely involved in reactions leading to the formation of sulfoxides and then sulfones, after which acyl-CoA dehydrogenases are engaged. Genes similar to *dsz*D were not found in the genome of *G. alkanivorans* strain 135.

Given the lack of accumulation of 2-HBP in the cultivation medium of *G. alkanivorans* 135 on DBT, as well as the strain’s ability to grow on hydroxylated aromatic compounds, it is hypothesized that after sulfur extraction from DBT, the subsequent transformation of metabolites occurs through the cleavage of aromatic rings. This process may involve acyl-CoA dehydrogenases. The involvement of acyl-CoA dehydrogenases in the cleavage of aromatic rings was reported by Alemayehu et al. [54]. In their work, the *ido*A gene, which is necessary for the degradation of fluoranthene by *Pseudomonas alcaligenes* PA-10, was discussed. The authors revealed that it is most closely related to an acyl-CoA dehydrogenase from *Novosphingobium aromaticivorans*. The authors of [55] highlighted the involvement of a novel acyl-CoA dehydrogenase in the degradation of indoleacetate, which is a conjugate of a six-membered benzene ring and a five-membered pyrrole aromatic ring.

As mentioned above, strain 135 is less efficient as a DBT degrader than other known *Gordonia* strains with this ability [36,37,56,57,58] (Table S5). In all the examples listed in Table S5 (except strain 135), the strains transform DBT via the 4S pathway (with *dsz* genes). We assume that the genetic mechanism used in the case of strain 135 for DBT degradation originally (in terms of evolution) involved a substrate other than DBT and, therefore, is not optimally adapted for DBT catabolism. Nevertheless, we have shown earlier [13] that DBT utilization is efficient enough not to consider it nonspecific.

### 3.5. Ultrafast Proteomics of G. alkanivorans Strain 135 Revealed DBT Desulfurization and Degradation of Aromatic Compounds

The goal of the proteomic analysis was to confirm the mechanism of DBT desulfurization by *G. alkanivorans* 135 suggested by the results of the transcriptome analysis. A list of differentially regulated genes observed in proteomic analysis can be found in Table S6.

**Figure 6:**
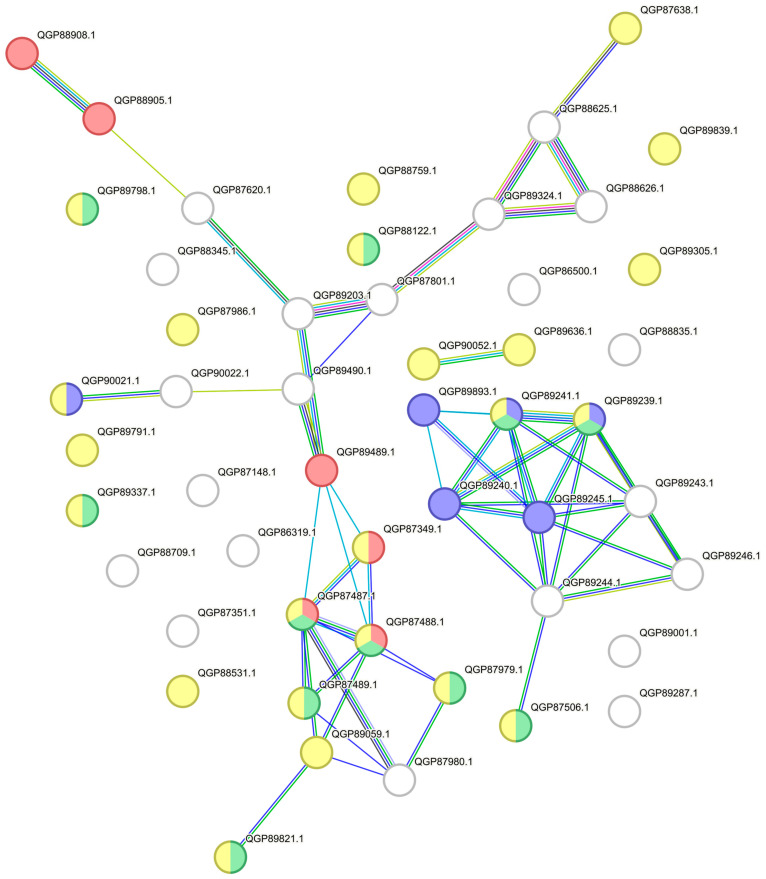
GO network from GO enrichment analysis of up-regulated proteins. The colors stand for the following GO terms: purple—propanoate metabolism (map00640), red—sulfur metabolism (map00920), green—monooxygenase (KW-0503), and yellow—oxidoreductase (KW-0560).

The proteins of the genes listed in Table 2 were found to be up-regulated, except for those of the *cat*C gene, the product of which was not identified in any of the samples. Gene ontology enrichment analysis of the up-regulated proteins demonstrated an enriched sulfur metabolism pathway. Among the up-regulated proteins, six proteins were found to belong to this pathway: QGP88908.1 sulfate ABC transporter substrate-binding protein, QGP88905.1 sulfate ABC transporter ATP-binding protein, QGP89489.1 sulfurtransferase, QGP87349.1 taurine dioxygenase, QGP87487.1 LLM class flavin-dependent oxidoreductase, and QGP87488.1 dimethyl sulfone monooxygenase SfnG. These proteins are marked in red in Figure 6. Other proteins previously discussed in this work were also found to be closely related to sulfur metabolism proteins, namely, QGP87489.1 SfnB family sulfur acquisition oxidoreductase, QGP89059.1 acyl-CoA dehydrogenase, and QGP87979.1 acyl-CoA dehydrogenase—which are associated with monooxygenase activity (marked green in Figure 6) and oxidoreductase activity (marked yellow in Figure 6)—and QGP89203.1 adenylyltransferase/sulfurtransferase MoeZ.

Furthermore, a set of proteins is presumably related to the degradation of aromatic compounds remaining after the desulfurization of DBT. The degradation of aromatic compounds starts with the oxidation to alcohols with mono- and dioxygenases. We observed several proteins of this nature, such as QGP87506.1 putative quinol monooxygenase, QGP89239.1 aromatic/alkene/methane monooxygenase hydroxylase/oxygenase subunit alpha, and QGP89241.1 aromatic/alkene monooxygenase hydroxylase subunit beta. Moreover, we detected two proteins associated with the formation and functioning of the iron–sulfur domain in protein complexes: QGP89244.1 iron–sulfur cluster assembly protein and QGP89240.1 2Fe-2S iron–sulfur cluster-binding protein. These proteins are the components of the cytochrome bc1 complex on which quinol oxidation occurs [59]. The next step of the aromatic compound degradation pathway is the oxidation of alcohols to aldehydes with alcohol dehydrogenase. Among the proteins related to propanoate metabolism (marked purple in Figure 6), we observed the two following enzymes of this class: QGP89893.1 zinc-dependent alcohol dehydrogenase family protein and QGP89245.1 NAD(P)-dependent alcohol dehydrogenase. Another component of this protein cluster is QGP89243.1, which belongs to the amidohydrolase family. A recent study showed that amidohydrolase plays a role in the degradation of aromatic compounds [60].

The DBT degradation pathway suggested by the results of RNA-seq analysis was confirmed by proteomics, which also revealed a cluster of proteins that is presumably responsible for further degradation of the substrate after desulfurization.

### 3.6. RNA-Seq and Proteomics Revealed Co-Expression of Enzymes Involved in DBT Degradation at Both the mRNA and Protein Levels

A comparison of differentially regulated proteins obtained by proteomic analyses and differentially expressed genes obtained by RNA-seq analysis (further referred to as differential features) showed only 17 overlapping features, which are presented in Table 3. Within this group of features, the FC values correlate with each other, with a Pearson correlation coefficient of 0.63, while no correlation is observed for the whole set of differential features (Pearson correlation coefficient of −0.24).

Thus, in a general comparison, the RNA-seq results do not fully agree with the proteomic results. This may be caused, for example, by a difference between mRNA maturation times and protein lifetimes, the presence of processes affecting protein biosynthesis, or post-translational modifications of proteins. However, the differential features that coincide in the proteomics and transcriptomics data indicate a major metabolic pathway of DBT degradation in *Gordonia* strains that is directly related to the culture conditions of the samples.

## 4. Conclusions

*Gordonia alkanivorans* strain 135 is not more capable of DBT degradation compared to 4S-desulfurizing *Gordonia*, but its DBT degradation process is unique. Since the metabolic pathway of DBT desulfurization in *G. alkanivorans* strain 135 does not involve genes of the *dsz* operon (4S-pathway), we investigated the possibility of DBT degradation via an alternative pathway through a combination of genomic, transcriptomic, and proteomic methods. Despite some differences in the data obtained via transcriptomic and proteomic analyses, it was shown that the *sfn*B and *tau*D genes and two acyl-dehydrogenase genes are significantly involved in the desulfurization process. Enzymatic processes involving these genes will be the subject of more precise and detailed investigations in our future studies.

## Figures and Tables

**Table 1:** Genes involved in sulfur metabolism whose expression was higher in the DBT sample than in the sodium sulfate sample (according to KEGG KO annotation).

KEGG KO	Gene	Name of Enzyme
K00641	*met*X	homoserine O-acetyltransferase/O-succinyltransferase [EC:2.3.1.31 2.3.1.46]
K01011	*sse*A	thiosulfate/3-mercaptopyruvate sulfurtransferase [EC:2.8.1.1 2.8.1.2]
K03119	*tau*D	taurine dioxygenase [EC:1.14.11.17]
K04091	*ssu*D	alkanesulfonate monooxygenase [EC:1.14.14.5]
K06881	*nrn*A	bifunctional oligoribonuclease and PAP phosphatase NrnA [EC:3.1.3.7 3.1.13.3]
K17228	*sfn*B	dimethylsulfone monooxygenase [EC:1.14.14.35]

**Table 2:** Genes selected for validation by RT-qPCR.

Gene Identifier and Name	log2 Fold Change DBT/Sulfate	*p*-Value	Gene Product
QGP87489.1 (*sfn*B)	−3.10521	5 × 10^−5^	SfnB family sulfur acquisition oxidoreductase
QGP87349.1 (*tau*D)	−5.78972	5 × 10^−5^	taurine dioxygenase TauD
QGP87979.1 (GKZ92_RS10190)	−2.6025	5 × 10^−5^	acyl-CoA dehydrogenase
QGP89059.1 (GKZ92_RS16320)	−2.83195	5 × 10^−5^	acyl-CoA dehydrogenase
QGP89675.1 (*cat*C)	−1.12099	0.005	muconolactone delta isomerase CatC
QGP89489.1 (*sse*A)	−2.94735	5 × 10^−5^	thiosulfate/3-mercaptopyruvate sulfurtransferase SseA
QGP89203.1 (*moe*Z)	−1.71539	5 × 10^−5^	adenylyltransferase/sulfurtransferase MoeZ

**Table 3:** Features that are differentially regulated in both proteomic and transcriptomic data. The MS1 proteomics and RNA-seq columns show the log2FC values for these features.

ID	Description	Expected Function	MS1 Proteomics	RNA-Seq
QGP89059.1	acyl-CoA dehydrogenase	DBT degradation	5.76	2.83
QGP87487.1	LLM class flavin-dependent oxidoreductase	unclassified metabolism	12.16	2.20
QGP89821.1	cadherin repeat domain-containing protein	cell adhesion	5.88	4.53
QGP89001.1	carbonic anhydrase	pH regulation in cell	5.21	4.96
QGP89490.1	DUF1416 domain-containing protein	unclassified metabolism	2.03	2.41
QGP87351.1	polyketide cyclase	polyketide synthesis	2.69	2.46
QGP87489.1	SfnB family sulfur acquisition oxidoreductase	DBT degradation	8.15	3.11
QGP87979.1	acyl-CoA dehydrogenase	DBT degradation	3.89	2.60
QGP87980.1	NtaA/DmoA family FMN-dependent monooxygenase	alkane degradation	12.16	2.60
QGP88626.1	50S ribosomal protein L21	universal ribosomal protein	1.44	2.20
QGP89324.1	50S ribosomal protein L24	universal ribosomal protein	1.87	2.42
QGP87986.1	nitroreductase family deazaflavin-dependent oxidoreductase	coenzyme F420 binding	2.63	2.47
QGP87620.1	bifunctional o-acetylhomoserine/o-acetylserine sulfhydrylase	methionine biosynthesis	2.62	3.76
QGP89377.1	mycofactocin-coupled SDR family oxidoreductase	unclassified metabolism	−6.27	−2.05
QGP87349.1	taurine dioxygenase	DBT degradation	12.16	5.79
QGP89489.1	sulfurtransferase	thiosulfate/3-mercaptopyruvate sulfurtransferase	2.00	2.95

## Data Availability

LC-MS1 data are available at proteomexchange.org (PXD058641). The genomic data are available in the GenBank database under accession number CP046257-CP046258 (BioProject PRJNA590456, BioSample SAMN13326465).

## Supplementary Materials

The following supporting information can be downloaded at https://www.mdpi.com/article/10.3390/biology14020188/s1: Table S1: Information about primers used for RT-qPCR; Table S2: Up-regulated genes observed in transcriptomic analysis; Table S3: Down-regulated genes observed in transcriptomic analysis; Table S4: Presence of *dsz* genes in the genomes of *G. alkanivorans* strains from the Genbank database; Table S5: Degradation of DBT by *Gordonia* strains; Table S6: List of differentially regulated genes observed in proteomic analysis; Figure S1: Enriched GO terms for up-regulated and down-regulated proteins obtained from MS1-based proteomic analysis.
