# Supplementary material for: Integrated Omics Approaches to Explore a New System of Genetic Control of Dibenzothiophene Desulfurization and Aromatic Ring Cleavage by Gordonia alkanivorans Strain 135
Source: Biology (Basel). 2025 Feb 12;14(2):188. doi: 10.3390/biology14020188 (PMC11852219; doi:10.3390/biology14020188)
Supplement: Supplementary file 1 [file biology-14-00188-s001.zip › biology-3454869-supplementary.pdf]

Table S1. Information about primers used for RT-qPCR.

**Gene product**	**Primer sequence**	**Amplicon size**	**E**	**R^2**
Reference gene candidates				
**16s rRNA**	16S(Gord)f GTGACGGTACCTGGAGAAGAGCAC 16S(Gord)r CGTATCGCCTGCACGCCTACA	166	2.02	0.992
CP046257.1_cds_QGP89257.1_3426 [gene=**aroA**] [locus_tag=GKZ92_17455] [protein=**3-phosphoshikimate 1-carboxyvinyltransferase**] [protein_id=QGP89257.1] [location=3822959..3824224]	aroA(Gordonia_135)RT60f CGGTTCCAAGTCCATCACCA aroA(Gordonia_135)RT164r AGCATCAGGTTGGTGTCTCG	105	1.93	0.996
CP046257.1_cds_QGP88962.1_3090 [gene=**ilvN**] [locus_tag=GKZ92_15745] [protein=**acetolactate synthase small subunit**] [protein_id=QGP88962.1] [location=complement(3470451..3470954)]	ilvN(Gordonia_135)RT266f TGATGGTCAAGGTCCGTTCG ilvN(Gordonia_135)RT429r GTAAGGATCGAGCATCCGCA	164	1.92	0.996
CP046257.1_cds_QGP90144.1_4407 [gene=**dinB**] [locus_tag=GKZ92_22365] [protein=**DNA polymerase IV**] [protein_id=QGP90144.1] [location=complement(4946788..4947996)]	dinB(Gordonia_135)RT528f CCTTCATCCTCTTCCGGTGC dinB(Gordonia_135)RT720r CTTTCCGGTTTCTACGCGAC	193	2.07	0.992
Genes of interest				
CP046257.1_cds_QGP87349.1_1303 [locus_tag=GKZ92_06655] [protein=**taurine dioxygenase**] [protein_id=QGP87349.1] [location=1455470..1456381]	tauD(Gordonia_135)RT541f TACTACCTCAAGCAGCGTCG tauD(Gordonia_135)RT607r CGAGTTCGGTGACTTCCCTG	67	2.02	0.997
CP046257.1_cds_QGP89489.1_3676 [locus_tag=GKZ92_18705] [protein=**sulfurtransferase**] [protein_id=QGP89489.1] [location=4140190..4141029]	tst(Gordonia_135)RT649f CTGTACGCGGACAAGGGATT tst(Gordonia_135)RT832r CTTCTCCGAGTTCGATCGGG	184	2.02	0.998
CP046257.1_cds_QGP89203.1_3370 [gene=**moeZ**] [locus_tag=GKZ92_17175] [protein=**adenylyltransferase/sulfurtransferase MoeZ**] [protein_id=QGP89203.1] [location=complement(3764793..3766001)]	moeZ(Gordonia_135)RT1057f GATCGCCCTACGGTCCTCTA moeZ (Gordonia_135)RT1206r GTAGACCGGCAACGTCTTGT	150	1.98	0.999

CP046257.1_cds_QGP89675.1_3897 [gene=**catC**] [locus_tag=GKZ92_19810] [protein=**muconolactone Delta-isomerase**] [protein_id=QGP89675.1] [location=complement(4379573..4379854)]	catC(Gordonia_135)RT82f AAGGCGTACTCCCAGGATCT catC(Gordonia_135)RT249r CGGTGTCACCTTGATGTCCA	168	1.97	0.995
CP046257.1_cds_QGP87489.1_1460 [locus_tag=GKZ92_07450] [protein=**SfnB family sulfur acquisition oxidoreductase**] [protein_id=QGP87489.1] [location=1627626..1628873]	*sfnB*(Gordonia_135)RT148f ; ATCGATCGCCTGTCGGAATC *sfnB*(Gordonia_135)RT321r ; GAGGCGCACCAGGTTTAGAT	274	2.03	0.995
CDS complement(2268230..2269429) /locus_tag="GKZ92_10185" /product="**acyl-CoA dehydrogenase**" /protein_id="QGP87979.1"	ACAD_79(sp.135)RT129f TTTGACCGCTGTGACTCTCG ACAD_79(sp.135)RT211r GGGCGAGATCGATGAGGAAA	83	2.03	0.995
CDS complement(3593029..3594252) /locus_tag="GKZ92_16320" /product="**acyl-CoA dehydrogenase**" /protein_id="QGP89059.1"	ACAD_59(Gordonia_135)RT478f AAGTACTACAGCACCGGCAG ACAD_59(Gordonia_135)RT601r CCCAGTCGTCGTGCTGAATG	124	2.02	0.988

Table S4. Presence of *dsz* genes in the genomes of *G. alkanivorans* strains from Genbank database

Strain name	Genbank assembly number	Assembly level	Presence of *dsz* genes	Presence of *sfn*B gene
YC-RL2	GCA_004011905.1	complete	-	-
GH-1	GCA_022591595.1	complete	+	+
152	GCA_029853935.1	contigs	-	+
134	GCA_029853965.1	contigs	+	+
12	GCA_029853985.1	contigs	+	+
142	GCA_029854005.1	contigs	-	+
132	GCA_029854035.1	contigs	-	+
133	GCA_029854045.1	contigs	-	+
144	GCA_029854075.1	contigs	-	+
96	GCA_029854095.1	contigs	-	+
129	GCA_029854135.1	contigs	-	+
IEGM 748	GCA_030057175.1	contigs	+	+
IEGM 1269	GCA_030057195.1	contigs	-	+
IEGM 1398	GCA_030058695.1	contigs	-	+
IEGM 1277	GCA_030063815.1	contigs	+	+
CGMCC 6845	GCA_000503935.1	contigs	-	+
NBRC 16433T	GCA_000225505.1	contigs	-	+
s104	GCA_000529675.1	contigs	-	+
**135 (this work)**	**GCA_009720185.1**	**complete**	**-**	**+**

* The strains with both dsz and sfnB are marked yellow. The strains with only sfnB (but no dsz) are marked green.

Table S5. Degradation of DBT by *Gordonia* strains

**Strain**	**Initial concentration of DBT**		**Degradation degree, %**	**Time of cultivation, days**	**Reference**
	mM	mg/L			
G. alkanivorans 1B	0.5	92	77	7	[37]
G. alkanivorans 135	0.2	37	45	6	[13]
G. rubripertincta MTCC 289	0.5	92	99	10	[56]
G. rubripertincta W3S5	0.2	37	99	2	[36]
G. alkanivorans RIPI90A	0.5	92	90	10	[57]
Gordonia sp. CYKS1	0.3	59	85	5	[58]

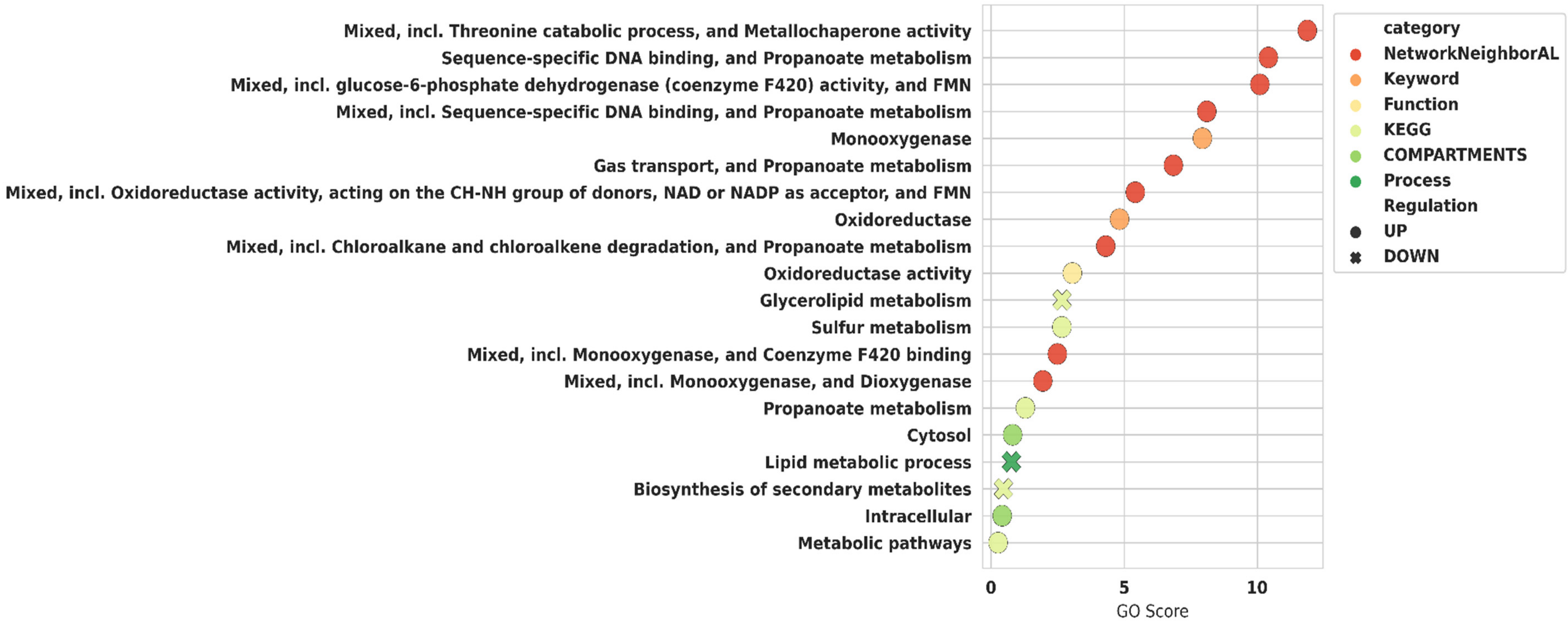

Figure S1. Enriched GO terms for up-regulated and down-regulated proteins obtained from MS1-based proteomic analysis.
